# Supplementary material for: Sirtuin 1 Is Required for Optimal Mammarenavirus Multiplication
Source: Viruses. 2026 Oct 2;18(10):1088. doi: 10.3390/v18101088 (PMC13709943; doi:10.3390/v18101088)
Supplement: Supplementary file 1 [file viruses-18-01088-s001.zip › Figure S1.pdf]

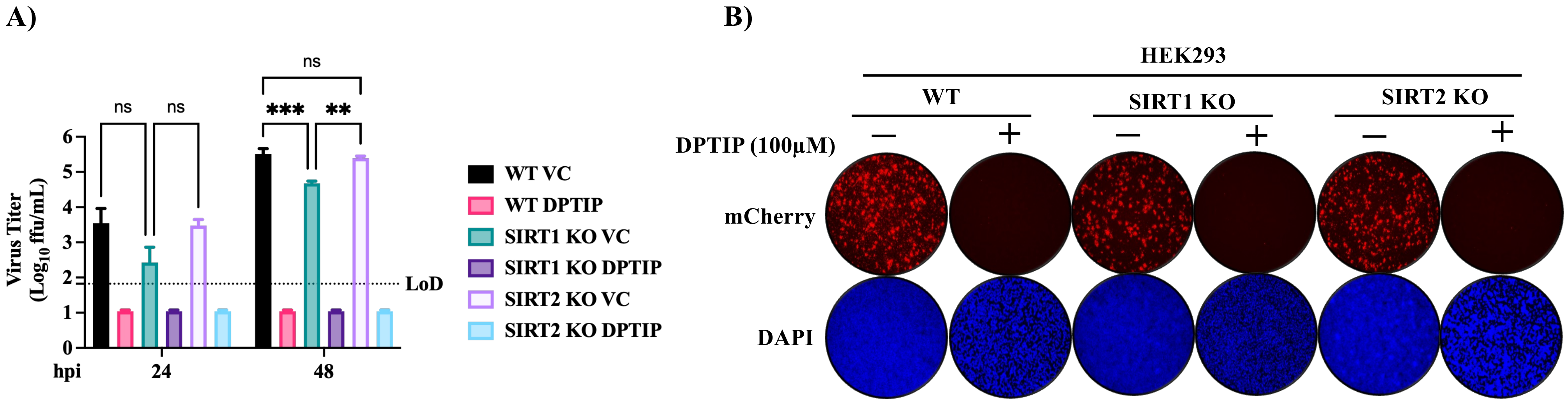

SF1. LCMV multiplication in SIRT1 and SIRT2 KO cells. A. HEK293 WT, SIRT1 or SIRT2 KO cells were seeded at 5×10^5 cells/well onto a 24-well plate, infected (MOI = 0.01) with rLCMV/mCherry and treated with DPTIP (100µM). At the indicated time points, CCSs were collected, and titers of infectious virus were determined by FFA using Vero E6 cells. **B**. At 48 hpi samples from (**A**) were fixed with 4% PFA and stained with DAPI and cell monolayer images (4× magnification) were acquired using a Keyence BZ-X710 microscope. Viral titers shown in panel **A**, correspond to mean ± SD from two biological replicates with each titration using technical triplicates. Results were analyzed using two-way ANOVA with Tukey's correction for multiple comparisons. Statistically significant values: ns $p > 0.05$, ** $p < 0.01$, *** $p < 0.001$.
